# c-Met and CREB1 are involved in miR-433-mediated inhibition of the epithelial–mesenchymal transition in bladder cancer by regulating Akt/GSK-3*β*/Snail signaling

**DOI:** 10.1038/cddis.2015.274

**Published:** 2016-02-04

**Authors:** X Xu, Y Zhu, Z Liang, S Li, X Xu, X Wang, J Wu, Z Hu, S Meng, B liu, J Qin, L Xie, X Zheng

**Affiliations:** 1 Department of Urology, First Affiliated Hospital, School of Medicine, Zhejiang University, Hangzhou, China

## Abstract

Emerging evidence has suggested that microRNAs (miRNAs) have an important role in tumor development and progression by regulating diverse cellular pathways. Here we describe the function and regulation network of miR-433 in bladder cancer (BCa). miR-433 is frequently downregulated in BCa tissues compared with adjacent non-cancerous tissues. Epigenetic mechanisms may be involved in the regulation of miR-433 expression. Enforced expression of miR-433 significantly inhibits proliferation, colony formation, migration, and invasion in BCa cells. In addition, miR-433 inhibits the epithelial–mesenchymal transition (EMT) in BCa cells by regulating *c-Met/Akt/GSK-3β/Snail* signaling pathway. Both *c-Met* and *CREB1* are downstream target genes of miR-433. *CREB1* can also indirectly regulate *c-Met/Akt/GSK-3β/Snail* signaling via *MITF*. Furthermore, *CREB1* expression is an independent prognostic factor for overall survival in patients with BCa. Finally, there appears to exist a reciprocal regulation between *c-Met* and miR-433/miR-409-3p. Taken together, this study reveals that miR-433-*c-MET/CREB1-Akt/GSK-3β/Snail* signaling is critical to EMT in BCa. Targeting the pathway described here may open up new prospects to restrict metastatic progression of BCa.

Bladder cancer (BCa) is the most common malignancy of the urinary tract. It is the 7th most common cancer in males and the 17th most common in females.^1^ It is an aggressive epithelial tumor characterized by a high rate of early systemic dissemination.^2^ Approximately one-third of BCa patients develop locally invasive or metastatic disease.^3^ Although numerous therapeutic strategies have been improved on and used in recent years, radical cystectomy remains the standard of care for invasive BCa,^4^ with a 5-year survival reported to be as low as 62% in the current literature.^5^ These data support continuing efforts to develop novel and more effective therapeutic strategies.

MicroRNAs (miRNAs) are a class of short (19–22 nucleotides) noncoding RNA sequences that suppress gene expression through sequence-specific binding with the 3′-untranslated region (3′-UTR) of a target mRNA.^6^ Emerging evidence reveals that disordered miRNA expression contributes to the initiation and progression of human BCa.^7^ We have previously identified a series of miRNAs, including miR-26a,^8^ miR-101,^9^ miR-124-3p,^10^ miR-320c,^11^ miR-409-3p,^12^ miR-490-5p,^13^ and miR-576-3p,^14^ that is involved in the proliferation, migration, and invasion of BCa cells. Nevertheless, the biological function of miRNAs in BCa and their downstream mechanisms remain elusive.

To further clarify the role of miRNAs in the pathogenesis of BCa, we have reviewed the published miRNA expression profiles in human BCa.^15, 16^ Based on these studies, miR-433 has captured our attention. miR-433 belongs to the DLK1-DIO3 miRNA cluster at 14q32.2 and is associated with the tumorigenicity and progression of gastric cancer,^17^ liver cancer,^18^ and myeloproliferative neoplasms.^19^ However, the exact role of miR-433 in BCa has not been documented.

In this study, we observed that miR-433 could inhibit the epithelial–mesenchymal transition (EMT) in BCa cells by regulating *Akt/GSK-3β/Snail* signaling. Both *c-Met* and *CREB1*, which were involved in the EMT process, were identified as direct target genes of miR-433. Furthermore, we described a reciprocal regulation between *c-Met* and miR-433/miR-409-3p.

## Results

### miR-433 is downregulated in BCa

**Figure 1:**
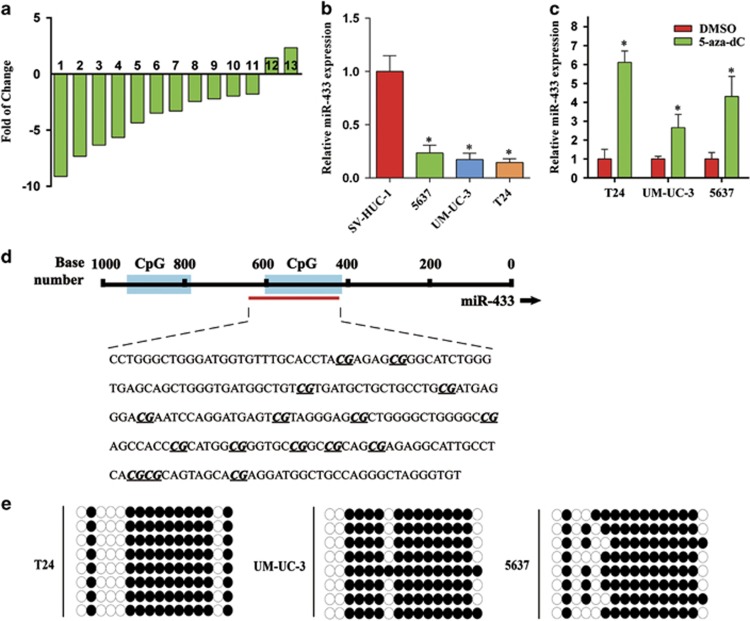
miR-433 is frequently downregulated in BCa and is regulated by DNA methylation. (**a**) The relative expression levels of miR-433 in individual 13 pairs of BCa tissues were presented as the fold change of miR-433 referred to the corresponding adjacent normal tissues. (**b**) The miR-433 levels in BCa cell lines (5637, UM-UC-3, and T24) were detected and compared with non-tumor urothelial cell line SV-HUC-1. (**c**) Demethylating agent 5-aza-dC stimulated the expression of miR-433 compared with DMSO-treated samples. (**d**) The regions analyzed by BSP are indicated. (**e**) Methylation profile in T24, UM-UC-3, and 5637. The open and filled circles represent the unmethylated and methylated CpGs, respectively. Eight clones from each cell line were analyzed. Error bars represent the S.E. obtained from three independent experiments; *P<0.05.

To evaluate the expression of miR-433 in BCa, quantitative real-time PCR (qRT-PCR) was performed in 13 pairs of clinical BCa tissues and adjacent non-cancerous tissues (the clinical characteristics of the patients are shown in Supplementary Table S1), as well as in three types of urinary BCa cell lines T24, UM-UC-3, and 5637. The expression level of miR-433 was generally lower in tumor tissue than in non-tumor tissue (Figure 1a, 11 out of 13 displayed a downregulation pattern). The examination of miR-433 in three different BCa lines also showed significant downregulation compared with SV-HUC-1 cell line (Figure 1b). These results are in agreement with a previously published data set,^15^ implying that miR-433 might have an important role in BCa progression.

Previous studies have suggested that methylation may participate in the regulation of miR-433 expression.^17, 18^ After treatment with 5-aza-2′-deoxycytidine (5-aza-CdR), a DNA methyltransferase inhibitor, the expression of miR-433 was significantly increased in the T24, UM-UC-3, and 5637 cell lines (Figure 1c). Two CpG islands were identified by the CpG Island Searcher program (http://www.urogene.org/methprimer/) from the 1000-bp upstream region of miR-433 (Figure 1d). The results of bisulfite-sequencing PCR (BSP) revealed that the T24, UM-UC-3, and 5637 cell lines showed a high methylation level (Figure 1e). These results supported the notion that DNA methylation might contribute to the deregulation of miR-433.

### Overexpression of miR-433 inhibits proliferation and colony formation in BCa cells

**Figure 2:**
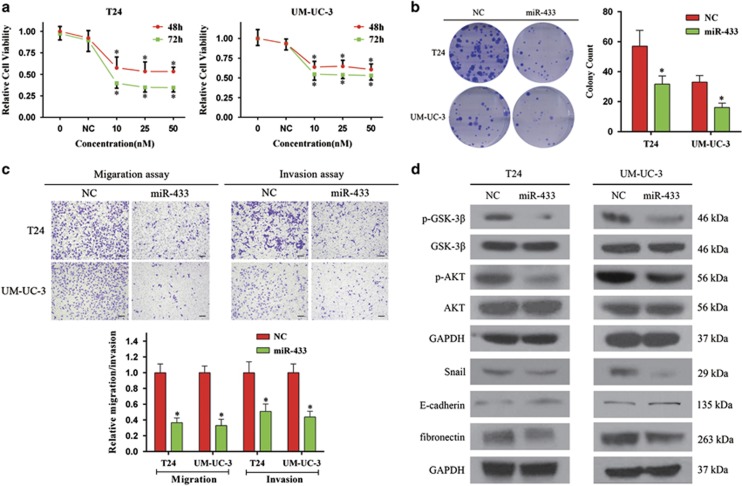
Effect of miR-433 in regulating BCa cells proliferation and motility. (**a**) CCK-8 assay. The relative cell viability of the miR-433-treated groups of T24 and UM-UC-3 cells were lower than that of NC-treated groups (cell viability of 0 nM was regarded as 1.0), respectively. (**b**) Colony-formation assay (representative wells were presented). The colony-formation rate was lower for miR-433 (50 nM)-transfected groups compared with NC (50 nM)-transfected groups. (**c**) Transwell assay (representative micrographs were presented). miR-433 (50 nM) impaired the motility of T24 and UM-UC-3 cells. (**d**) Western blot analysis. miR-433 (50 nM) inhibited EMT and *AKT/GSK-3β* signaling-related proteins in T24 and UM-UC-3 cells. Error bars represent the S.E. obtained from three independent experiments; *P<0.05. Scale bars=100 *μ*m.

T24 and UM-UC-3 cells were transfected with miR-433 mimics. The ectopic expression of miR-433 was confirmed by qRT-PCR (Supplementary Figure S1). Although there was no significant dosage effect, miR-433 suppressed the growth of cultured BCa cells at different concentrations and time points (Figure 2a). In parallel, miR-433 also had a marked impact on the colony-forming ability of BCa cells. The colony-formation capacity of miR-433 transfected cells was much lower than in cells transfected with NC (Figure 2b). These results suggested that miR-433 could regulate proliferation in BCa cells.

### Overexpression of miR-433 inhibits cell motility and induces the EMT

Next, we examined whether miR-433 had any effect on migration and invasion capacity. A wound-healing assay indicated that the overexpression of miR-433 in UM-UC-3 cells was associated with a retardation of wound closure compared with the control group (Figure 4a). A migration chamber assay was undertaken to verify the role of miR-433 in cell motility. As shown in Figure 2c, miR-433 significantly suppressed the migration and invasion of T24 and UM-UC-3 cells.

As EMT is a key event in cancer invasion and metastasis, the effect of miR-433 on EMT was analyzed by detecting the protein levels of EMT markers. As shown in Figure 2d and Supplementary Figures S2 and S3, overexpression of miR-433 increased the expression of *E-cadherin* (an epithelial marker) but decreased *fibronectin*, *Vimentin* (mesenchymal markers), and *Slug* and *Snail* (EMT-related transcription factors). We further explored the underlying mechanism for miR-433-suppressed EMT. It has been well documented that the activation of *AKT* increases the nuclear expression and transcriptional activity of *Snail* by inhibitory phosphorylation of *GSK-3β*, thereby triggering cell migration and EMT.^20^ We detected *AKT/GSK-3β* signaling-related proteins by western blot analysis and found that the expression of phosphorylated *Akt* and phosphorylated *GSK-3β* (inactive form) were decreased in miR-433-transfected cells (Figure 2d). These results indicated that the overexpression of miR-433 could suppress the EMT phenotype of T24 and UM-UC-3 cells by regulating *Akt/GSK-3β/Snail* signaling.

### *CREB1* and *c-Met* are direct targets of miR-433

**Figure 3:**
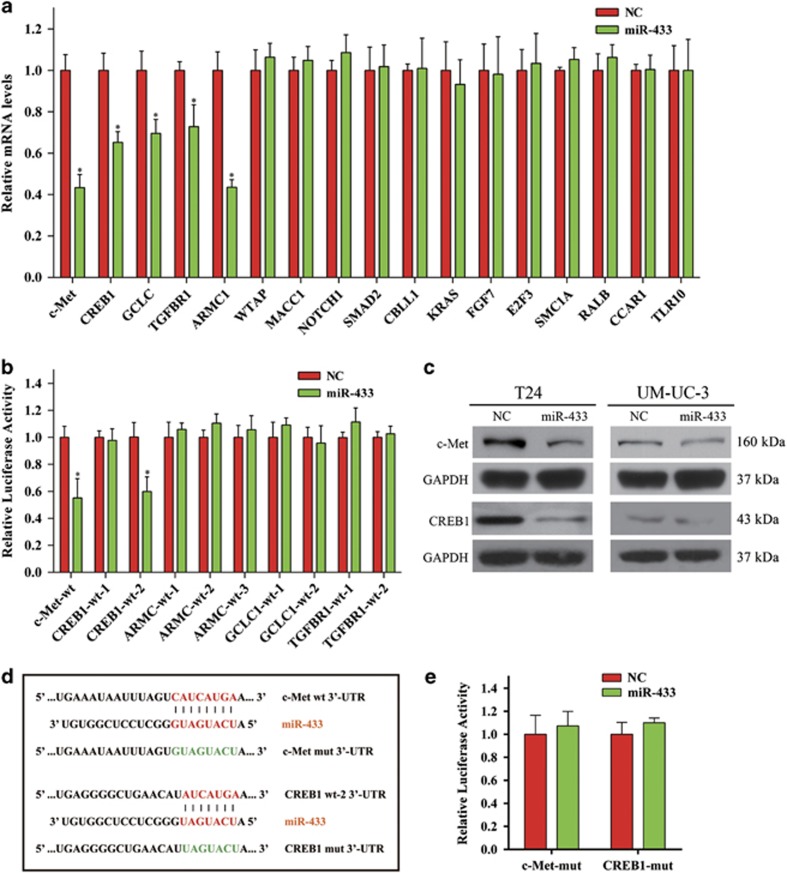
*c-Met* and *CREB1* are direct targets of miR-433. (**a**) qRT-PCR analysis. Decreased expression of *c-Met, CREB1, GCLC, TGFßR1*, and *ARMC1* were observed in miR-433-transfected UM-UC-3 cells. (**b**) Dual-luciferase reporter assay. miR-433 significantly suppressed the luciferase activity of vectors that carried 3′-UTRs of *c-Met* and *CREB1* but not *GCLC*, *TGFβR1*, and *ARMC1*. (**c**) Western blot analysis confirmed that miR-433 inhibited the endogenous expression of *c-Met* and *CREB1*. (**d**) The miR-433-targeting sites in 3′-UTRs of *c-Met* and *CREB1* were mutated. (**e**) Dual-luciferase reporter assay. The luciferase activity of the mutated vectors were unaffected by the transfection of miR-433. Error bars represent the S.E. obtained from three independent experiments; *P<0.05.

We used two methods to screen for potential direct targets of miR-433: bioinformatics prediction (http://www.targetscan.org/) and gene expression microarray analysis. Based on the results of bioinformatics prediction and microarray analysis (Supplementary Table S2), we identified several candidate target genes and subsequently tested the expression of these genes after treatment with miR-433 mimics by qRT-PCR. A decrease in the expression of *c-Met, CREB1*, *GCLC, TGFβR1*, and *ARMC1* was observed in miR-433-transfected BCa cells (Figure 3a).

We then performed luciferase reporter assays to examine the direct interaction between miR-433 and the 3′-UTRs of the five genes (*c-Met*, *CREB1*, *GCLC*, *TGFβR1*, and *ARMC1*). We cloned the 3′-UTRs into pmirGLO Dual-Luciferase miRNA Target Expression Vectors. As shown in Figure 3b, overexpression of miR-433 significantly decreased the relative luciferase activity of *c-Met* and *CREB1* but had no effect on the other genes. Western blot analysis revealed a reduced protein expression of *c-Met* and *CREB1* in miR-433-transfected cells (Figure 3c). To verify that miR-433 directly binds to the 3′-UTRs of *c-Met* and *CREB1*, we mutated the miR-433-targeting sites (Figure 3d). As expected, the luciferase activity of the mutated vectors was unaffected by the transfection of miR-433 (Figure 3e). Collectively, these data supported the notion that *CREB1* and *c-Met* were direct downstream targets of miR-433.

### Repression of *c-Met* inhibits cell motility and induces EMT

**Figure 4:**
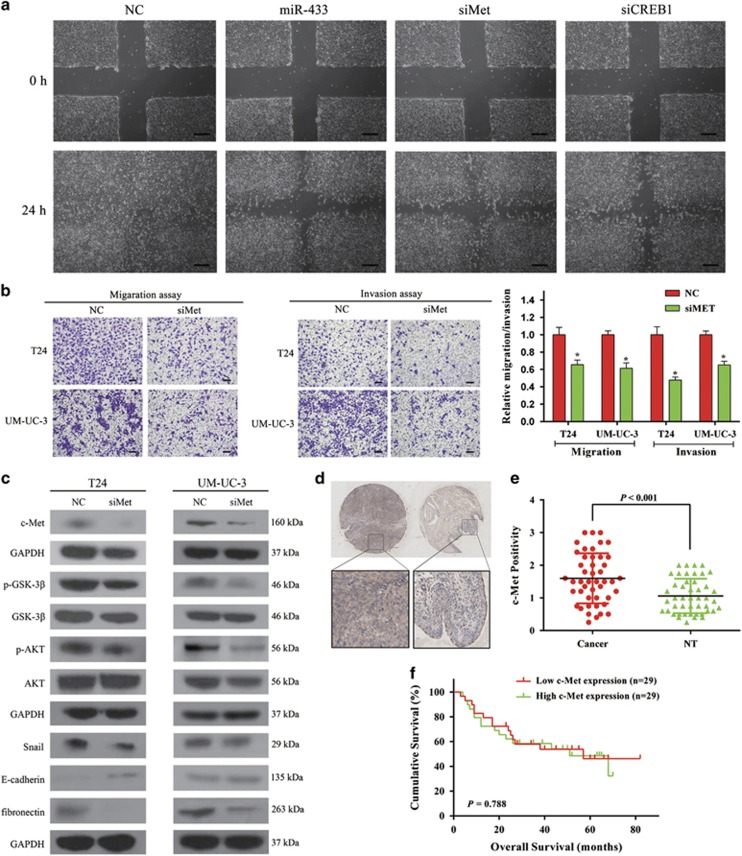
Knockdown of *c-Met* suppresses BCa cells motility. (**a**) UM-UC-3 cells were transfected with NC, miR-433 mimics, siMet, and siCREB1, respectively. Wound-healing assay was performed with a 24-h recovery period. (**b**) Transwell assay (representative micrographs were presented). siMet impaired the motility of T24 and UM-UC-3 cells. (**c**) siMet inhibited EMT and *AKT/GSK-3β* signaling-related proteins in T24 and UM-UC-3 cells. (**d**) Representative images of IHC staining of TMA. *c-Met* showed a membranous and cytoplasmic location. (**e**) Statistical analysis indicated that the expression level of *c-Met* protein in BCa tissues was significantly higher than that in adjacent non-tumor tissues. (**f**) Kaplan–Meier survival analysis. The protein expression of *c-Met* was not associated with the overall survival rate in BCa patients. Error bars represent the S.E. obtained from three independent experiments; *P<0.05. Scale bars=100 *μ*m.

We previously reported that knockdown of *c-Met* by siMet inhibited migration and invasion in BCa cells,^12^ but the underlying mechanism was not explored. Considering the importance of *c-Met/AKT/GSK-3β/Snail* signaling in EMT and metastasis,^21, 22^ we used western blottings to examine the expression of several proteins involved in this signaling pathway. Decreased expression of phosphorylated *Akt*, phosphorylated *GSK-3β*, *fibronectin*, and *Snail*, as well as increased expression of *E-cadherin*, were observed in siMet-transfected cells (Figure 4c). Accordingly, the migration and invasion of BCa cells were significantly suppressed (Figures 4a and b). These results phenocopied the effect of miR-433 on cell motility and EMT.

We further investigated the expression patterns and subcellular localizations of *c-Met* protein in BCa tissues and adjacent non-tumor tissues by immunohistochemical (IHC) analysis. *c-Met* showed a membranous and cytoplasmic location (Figure 4d). Statistical analysis indicated that the expression level of *c-Met* protein in BCa tissues was significantly higher than in adjacent non-tumor tissues (*P*<0.001, Figure 4e). To evaluate the prognostic role of *c-Met*, a survival analysis was executed. Kaplan–Meier survival curves indicated that the protein expression of *c-Met* was not associated with the overall survival rate of BCa patients (*P*=0.788, Figure 4f).

### Repression of CREB1 inhibits cell proliferation and induces EMT

**Figure 5:**
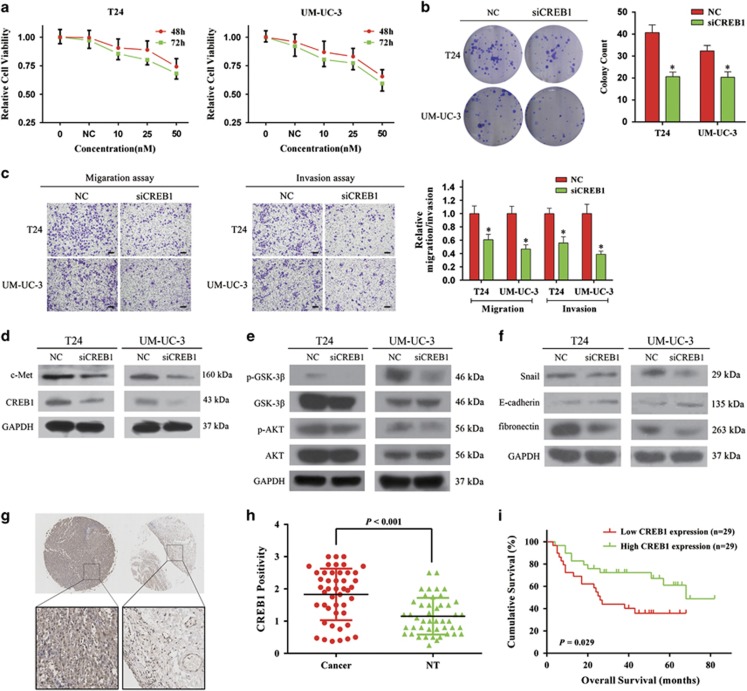
Knockdown of *CREB1* suppresses BCa cell proliferation and motility. (**a**) CCK-8 assay. The relative cell viability of the siCREB1-treated groups of T24 and UM-UC-3 cells were lower than that of NC-treated groups (cell viability of 0 nM was regarded as 1.0), respectively. (**b**) Colony-formation assay (representative wells were presented). The colony-formation rate was lower for siCREB1-transfected groups compared with NC-transfected groups. (**c**) Transwell assay (representative micrographs were presented). siCREB1 impaired the motility of T24 and UM-UC-3 cells. (**d**) siCREB1 inhibited *CREB1* and *c-Met* proteins. (**e**) siCREB1 inhibited *AKT/GSK-3β* signaling-related proteins. (**f**) siCREB1 inhibited EMT-related proteins. (**g**) Representative images of IHC staining of TMA. *CREB1* localized in the nucleus and cytoplasm. (**h**) Statistical analysis indicated that the expression level of *CREB1* protein in BCa tissues was significantly higher than that in adjacent non-tumor tissues. (**i**) Kaplan–Meier survival analysis. The protein expression of *CREB1* was significantly associated with the overall survival rate in BCa patients. Error bars represent the S.E. obtained from three independent experiments; *P<0.05. Scale bars=100 *μ*m.

The exact role of *CREB1* in BCa is unclear. We first analyzed the effect of *CREB1* on cell proliferation. The CCK-8 assay showed that knockdown of *CREB1* by siCREB1 inhibited the growth of T24 and UM-UC-3 cells at different concentrations and time points (Figure 5a). In parallel, the colony-forming ability of BCa cells was decreased. The colony-formation capacity of siCREB1-transfected cells was much worse than in cells transfected with NC (Figure 5b). Next, we explored the role of *CREB1* in the migration and invasion of BCa cells. The wound-healing assay indicated that the downregulation of *CREB1* in UM-UC-3 cells led to a retardation of wound closure compared with the control group (Figure 4a). A migration chamber assay was also performed and revealed that migration and invasion were suppressed in siCREB1-transfected cells (Figure 5c). These data suggested that *CREB1* could regulate BCa cell proliferation and motility.

We were curious as to whether *CREB1* also participated in EMT, which has not been well established. It has been reported that *CREB1* can directly bind to the *MITF* promoter region and stimulate *MITF* transcription.^23^ On the other hand, *c-MET* transcription can be directly regulated by *MITF*.^24^ Thus, we speculated that *CREB1* might indirectly regulate the expression of *c-Met*. This hypothesis was supported by western blot analysis, which showed that knockdown of *CREB1* by siCREB1 reduced *c-Met* protein expression (Figure 5d). As expected, downregulation of *CREB1* also inhibited the *AKT/GSK-3β/Snail* pathway. Decreased expression of phosphorylated *Akt*, phosphorylated *GSK-3β*, *fibronectin*, and *Snail*, as well as increased expression of *E-cadherin*, were detected in siCREB1-transfected cells (Figures 5e and f). Collectively, these data implied that the repression of *CREB1* could suppress the EMT phenotype of T24 and UM-UC-3 cells at least partially by regulating *c-Met/Akt/GSK-3β/Snail* signaling.

We further examined the expression patterns and subcellular localizations of *CREB1* proteins in BCa tissues and adjacent non-tumor tissues by IHC analysis. *CREB1* localized in the nucleus and cytoplasm (Figure 5g). Statistical analysis indicated that the expression level of *CREB1* protein in BCa tissues was significantly higher than in adjacent non-tumor tissues (*P*<0.001, Figure 5h). Kaplan–Meier survival curves indicated that the protein expression of *CREB1* was significantly associated with the overall survival rate of BCa patients (*P*=0.029, Figure 5i). Finally, in a multivariate Cox model including sex, age, tumor grade, T stage, lymph node status, *c-Met* expression, and *CREB1* expression, we found that the overexpression of *CREB1* expression was a good independent prognostic factor for overall survival in patients with BCa (*P*=0.039, Supplementary Table S3).

### *CREB1* can indirectly regulate the expression of *c-Met* via *MITF*

**Figure 6:**
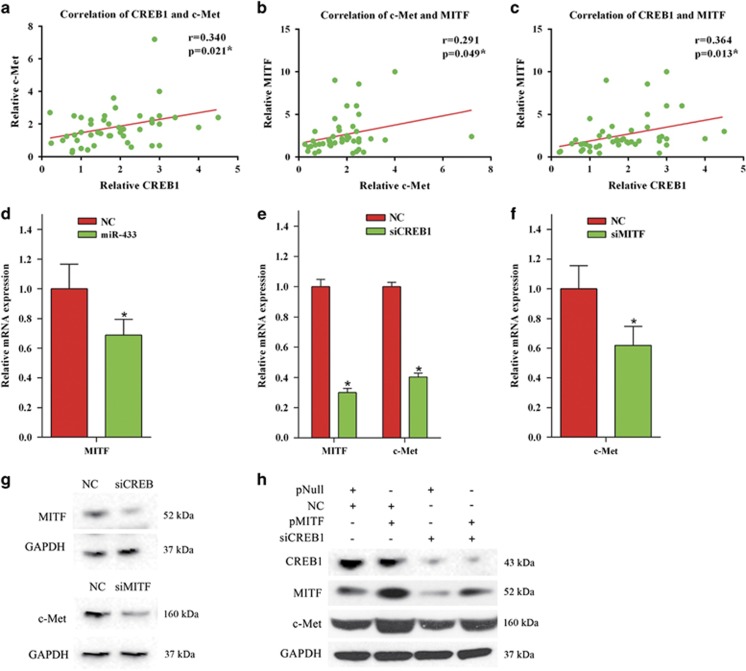
*CREB1* can indirectly regulate the expression of *c-Met* via *MITF*. (**a**) Expression of *CREB1* was significantly positively correlated with *c-Met* (*r*=0.340, *P*=0.021) in BCa tissues. (**b**) *c-Met* was significantly positively correlated with *MITF* (*r*=0.291, *P*=0.049). (**c**) *CREB1* was significantly positively correlated with *MITF* (*r*=0.364, *P*=0.013). (**d**) Overexpression of miR-433 in UM-UC-3 cells inhibited the mRNA level of *MITF*. (**e**) Repression of *CREB1* in UM-UC-3 cells inhibited the mRNA level of *MITF* and *c-Met*. (**f**) Inhibition of *MITF* suppressed the mRNA level of *c-Met*. (**g**) At the protein level, repression of *CREB1* and *MITF* in UM-UC-3 cells inhibited the *MITF* and *c-Met*, respectively. (**h**) Overexpression of *MITF* was able to, at least partially, rescue the level of *c-Met* in the presence of siCREB1. Error bars represent the S.E. obtained from three independent experiments; *P<0.05.

We compared the expression of *CREB1*, *c-Met*, and *MITF* in BCa tissues. As shown in Figures 6a and c, *CREB1* was significantly positively correlated with c-Met (*r*=0.340, *P*=0.021) and *MITF* (*r*=0.364, *P*=0.013). A significantly positive correlation between *c-Met* and *MITF* (*r*=0.291, *P*=0.049) was also observed (Figure 6b). Furthermore, either overexpression of miR-433 or repression of *CREB1* in UM-UC-3 cells inhibited the mRNA level of *MITF* (Figures 6d and e). On the other hand, inhibition of *MITF* suppressed the mRNA level of *c-Met* (Figure 6f). At the protein level, repression of *CREB1* and *MITF* also inhibited the *MITF* and *c-Met*, respectively (Figure 6g). Finally, we performed a rescue experiment, which showed that *MITF* overexpression was able to, at least partially, rescue the level of *c-Met* in the presence of siCREB1 (Figure 6h).

### miR-409-3p is involved in miR-433-mediated inhibition of cell motility

**Figure 7:**
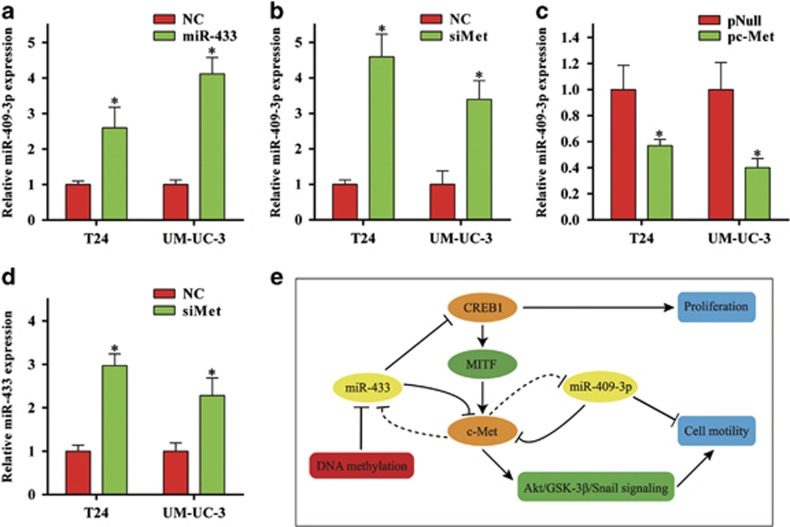
miR-409-3p is involved in miR-433-mediated inhibition of cell motility. (**a**) Overexpression of miR-433 significantly increased the expression of miR-409-3p. (**b**) Knockdown of *c-Met* increased the expression of miR-409-3p. (**c**) Upregulation of *c-Met* by an overexpression vector (pc-Met) reduced the expression of miR-409-3p. (**d**) Knockdown of *c-Met* increased the expression of miR-433. (**e**) Schematic diagram showing the signaling network in which miR-433 is involved. Error bars represent the S.E. obtained from three independent experiments; *P<0.05.

A previous study has suggested that *c-Met* could regulate the 14q32.2 miRNA cluster (e.g., the DLK1-DIO3 miRNA cluster),^25^ which includes miR-433 and miR-409-3p. On the other hand, our previous study^12^ and this study indicated that miR-409-3p and miR-433 could directly regulate *c-Met*. Thus, we speculated that there might be reciprocal regulation between miR-433/miR-409-3p and *c-Met*. Consistent with this assumption, downregulation of *c-Met* by siMet increased the expression of miR-433 and miR-409-3p (Figures 7b and d), whereas upregulation of *c-Met* by an overexpression vector (pc-Met) reduced the expression of miR-409-3p (Figure 7c). Overexpression of miR-433 could also significantly increase the expression of miR-409-3p (Figure 7a), at least partially, by inhibiting the expression of *c-Met*. A genetic regulatory network is depicted in Figure 7e and summarizes the key findings of our study.

## Discussion

Recent evidence suggests that the deregulation of miRNAs has an important role in tumorigenesis and progression in BCa and other malignancies. Numerous studies have characterized the miRNA signatures of BCa, but the exact role of miRNA dysregulation in the pathogenesis of BCa remains elusive. In this study, the expression of miR-433 was significantly lower in BCa tissues than in adjacent non-cancerous tissues. Furthermore, gain-of-function analyses showed that miR-433 suppressed the proliferation, migration, and invasion of BCa cells. These findings have highlighted the tumor suppressor role of miR-433 in BCa.

Human miR-433, located at chromosomal region 14q32.2, belongs to the DLK1-DIO3 genomic imprinted miRNA cluster and functions as a tumor suppressor in several different types of human cancers.^17, 18, 19^ Epigenetic programming has an important role in the regulation of miR-433. Guo *et al.*^17^ and Yang *et al.*^18^ showed that miR-433's surrounding CpG islands were hypermethylated, and that treatment with the DNA methylation inhibitor 5-aza-CdR could induce miR-433 expression. Similarly, the results of our study suggested that epigenetic mechanisms might be involved in the regulation of miR-433 expression in BCa. Furthermore, Saito *et al.*^26^ showed that the expression of *miR-127*, the paired gene that is clustered with miR-433, is regulated epigenetically in BCa cells. Collectively, the deregulation of miR-433 in human cancers may be partly attributed to the epigenetic modification of chromatin.

Although several studies have characterized the function and mechanism of miR-433, the molecular basis of the miR-433-mediated antitumor effect is still not well elucidated. In this study, two direct targets of miR-433, namely, *c-Met* and *CREB1*, were identified and experimentally verified. *c-Met* is a well-characterized oncogene and is frequently overexpressed in a variety of human cancers.^27^ We have previously confirmed that miR-409-3p can inhibit the migration and invasion of BCa cells by targeting *c-Met*.^12^ In this study, we found that the miR-433 downregulation is a new mechanism responsible for the abnormal activation of *c-Met*. We further showed that *c-Met* has a crucial role in miR-433-induced inhibition of EMT. Reduction of *c-Met* by miR-433 suppressed the activating phosphorylation of *AKT* and the inhibitory phosphorylation of *GSK-3β*, leading to the nuclear accumulation of *Snail* and increased expression of *E-cadherin*. Interestingly, we observed that *c-Met* could reversely regulate the expression of miR-409-3p and miR-433, which was in accordance with the findings of a previous study that reported *c-Met* could regulate the 14q32.2 miRNA cluster.^25^ This reciprocal regulation, therefore, maximizes the activation of the *c-Met* signaling pathway in BCa cells in response to HGF stimulation.

As a transcription factor, *CREB1* controls numerous downstream genes and has been implicated in cancer cell migration and invasion in various malignancies.^18, 28^ Nevertheless, the exact role of *CREB1* in EMT remains elusive. In this study, we showed that downregulation of *CREB1* could suppress the *AKT/GSK-3β/Snail* pathway at least partially via decreasing the expression of *c-Met*. The molecular mechanisms underlying *CREB1*-induced inhibition of *c-Met* are not well defined in the literature. As *CREB1* can directly promote the transcription of *MITF*, which directly regulates the expression of *c-Met*,^29^ we speculate that *MITF* may be involved in *CREB1*-mediated activation of *c-Met/AKT/GSK-3β/snail* signaling. In addition, *CREB-VEGF* signaling has been implicated in the process of EMT in prostate cancer.^30^ All these data support the notion that *CREB1* has a crucial role in EMT and metastasis. Furthermore, *CREB1* is also a direct target of miR-433; thus, the expression of *c-Met* is directly and indirectly regulated by miR-433 in BCa cells. Finally, it should be noted that a single miRNA can regulate the mRNA transcripts of hundreds of target genes, and we cannot rule out the possibility that signaling pathways mediated by other targets, apart from *c-Met* and *CREB1*, may have a role in miR-433-mediated inhibition of EMT.

In survival analyses, a significantly better overall survival was observed in patients with high *CREB1* expression, which seems contradictory to the tumor-promoting role of *CREB1*. Similarly, Liu *et al.*^31^ reported that overexpression of *p-CREB* (Ser133) and *CREB* correlated with a favorable survival in squamous cell lung carcinoma. They speculated that overexpression of *CREB* might mainly occur in early-stage tumors. Based on this hypothesis, we reviewed the existing BCa microarray data sets deposited in the Oncomine database.^32^ Seven data sets that compared superficial BCa with infiltrating BCa were identified from Oncomine. As shown in Supplementary Figure S4, the *CREB1* expression in the superficial samples was generally higher than that in the infiltrating samples in six of seven data sets, of which three data sets have statistically significant differences. These results implied that *CREB1* might have a more important role in the progression of early-stage BCa. Nevertheless, as the size of our patient cohort is limited, further prognostic study is warranted to confirm the findings of our study. Of note, Chhabra *et al.*^33^ and Yu *et al.*^34^ showed that overexpression of *CREB1* predicted an unfavorable prognosis in patients with breast cancer and hepatocellular carcinoma, respectively. The prognostic effect of *CREB*, therefore, seems to be cancer-type specific and context specific.

In conclusion, we report the following new findings: (i) miR-433 is frequently downregulated in BCa at least partially due to altered DNA methylation; (ii) miR-433 functions as a tumor suppressor in BCa cells; (iii) *c-Met* and *CREB1* are identified as downstream target genes of miR-433; (iv) miR-433 inhibits EMT in BCa cells by regulating *c-Met/Akt/GSK-3β/Snail* signaling; (v) *CREB1* can also indirectly regulate *c-Met/Akt/GSK-3β/Snail* signaling via *MITF*; (vi) *CREB1* expression is an independent prognostic factor for overall survival in patients with BCa; and (vii) a reciprocal regulation between *c-Met* and miR-433/miR-409-3p may exist. Our study underscores the important role of miR-433 in BCa progression and we expect that our findings on the signaling axis of miR-433-*CREB1/c-Met-Akt/GSK-3β/Snail* will provide useful information for the development of more effective and promising therapies against BCa.

## Materials and Methods

### Reagents and transfection

The miR-433 mimic (named miR-433, sense; 5′-AUCAUGAUGGGCUCCUCGGUGU-3′) and the negative control duplex (named NC, sense; 5′-ACUACUGAGUGACAGUAGA-3′) with no significant homology to any known human sequences were used for gain-of-function studies. The small interfering RNA targeting human *c-Met* mRNA (named siMet, sense; 5′-GGAGGUGUUUGGAAAGAUAdTdT-3′), *CREB1* mRNA (named siCREB1, sense; 5′-GAGAGAGGUCCGUCUAAUGdTdT-3′), and *MITF* mRNA (named siMITF, sense; 5′-AGCAGUACCUUUCUACCACdTdT-3′) were used for the RNA interference study. The RNA duplexes were chemically synthesized by GenePharma, Shanghai, China. Oligonucleotide transfection was performed using Lipofectamine 2000 reagents (Invitrogen, Carlsbad, CA, USA) in accordance with the manufacturer's protocol.

### Cell lines and cell culture

The human BCa cell lines T24, UM-UC-3, and 5637, as well as one normal bladder cell line SV-HUC-1, were purchased from the Shanghai Institute of Cell Biology, Shanghai, China. These cell lines were maintained in RPMI 1640 medium containing 10% heat-inactivated fetal bovine serum (FBS) under a humidified atmosphere of 5% CO_2_ at 37 °C.

### Human clinical samples

Paired BCa tissues and adjacent non-neoplastic bladder mucosal tissues were obtained from patients undergoing radical cystectomy. The samples were collected between January 2011 and October 2013 at the First Affiliated Hospital of Zhejiang University, after informed consent and Ethics Committee's approval. The clinical data of the patients has been listed in our previous studies.^11^ Tissue samples were snap frozen in liquid nitrogen until RNA extraction.

### RNA isolation and qRT-PCR

miRNA was extracted from clinical samples and cultured cell lines using RNAiso for Small RNA (Takara, Dalian, China) and reverse transcribed using the One Step PrimeScript miRNA cDNA Synthesis Kit (Takara). Total RNA was isolated with RNAiso Plus (Takara) and transcribed into cDNA using the PrimeScript RT Reagent Kit (Takara). qRT-PCR was performed using an ABI 7500 FAST Real-Time PCR System (Applied Biosystems, Carlsbad, CA, USA) and a SYBR Green PCR Kit (Takara, Dalian, China). The relative expression level of miRNA or mRNA was quantified with the 2^−ΔΔCt^ method after normalization with the endogenous reference U6 small nuclear RNA or *GAPDH*, respectively. The primers are shown in Supplementary Table S4.

### Dual-luciferase reporter assay

Oligonucleotide pairs that contained the desired miR-433 target region or mutant target region were designed and ordered from Sangon, Shanghai, China. After annealing, these double-stranded segments were inserted into pmirGLO Dual-Luciferase miRNA Target Expression Vector (Promega, Madison, WI, USA), between the *Sac*I and *Sal*I sites. The insertions were verified by sequencing. HEK293T cells were seeded in 24-well plates and co-transfected with 50 nM miR-433 or NC and 100 ng reporter pmirGLO. The relative luciferase activity was measured by the Dual-Luciferase Reporter Assay System (Promega) 48 h after transfection.

### Cell growth/cell viability assay

T24 or UM-UC-3 cells were plated in 96-well plates with ~4 × 10^3^ cells per well. After overnight incubation, the cells were transfected with the RNA duplex (miR-433, siCREB1, or NC) for 2–3 days with concentrations ranging from 10 to 50 nM. At different time points, the medium was removed and WST-8 (Dojindo Laboratories, Kumamoto, Japan) was added to each well. After the 96-well plate was incubated at 37 °C for 1 h, the absorbance of the solution was measured spectrophotometrically at 450 nm with a MRX II absorbance reader (Dynex Technologies, Chantilly, VA, USA).

### Colony-formation assay

Cells were trypsinized to a single cell suspension 24 h after transfection with 2'-*O*-methyl-modified duplexes (50 nM). Next, the cells were seeded in six-well plates (500 cells per well) and maintained under standard culture conditions for 2 weeks. Colony counts were performed after the colonies were fixed with absolute methanol and stained with 0.1% crystal violet.

### Wound-healing assays

After transfection, the cells were grown to 100% confluence in six-well plates. A micropipette tip was used to make a cross wound and wound healing was observed after 24 h. Photographs were taken under phase-contrast microscopy (Olympus, Tokyo, Japan) immediately or 24 h after wounding.

### Cell migration and invasion assay

The cell migration and invasion assay was performed using transwell chambers (Millipore, Boston, MA, USA). For the invasion assay, the inserts were coated with Matrigel (BD Bioscience, Franklin Lakes, NJ, USA) on the upper surface. After transfection, 8 × 10^4^ cells were suspended in 0.2 ml serum-free medium and added to the inserts. Next, 0.6 ml RPMI-1640 medium with 10% FBS was added to the lower compartment as a chemoattractant. After incubation at 37 °C for 24 h, the cells on the upper surface of the membrane were carefully removed using a cotton swab and cells on the lower surface were fixed with 100% methanol and stained with 0.1% crystal violet. Five visual fields of × 200 magnification of each insert were randomly selected and counted under a light microscope (Olympus).

### DNA methylation analysis and 5-aza-CdR treatment

BSP was performed as previously described.^35^ Following bisulfite conversion, the CpG islands of miR-433 were amplified by PCR using the primers 5′-TTTGGGTTGGGATGGTGTTTG-3′ (forward) and 5′-ACACCCTAACCCTAACAACCATCC-3′ (reverse). The PCR products were cloned into the pUC18 T-vector. After bacterial amplification, eight clones were subjected to DNA sequencing (Sangon). T24 and UM-UC-3 cells were treated with 5 *μ*M 5-aza-CdR (Sigma, St Louis, MO, USA) for 4 days. RNA was extracted and analyzed for the expression of miR-433.

### IHC staining

Tissue microarrays (TMAs) containing 46 cases with paired tumor and non-tumor tissues and 13 cases without corresponding non-tumor tissues were analyzed in this study. TMA was obtained from Xinchao Biotech, Shanghai, China. Paraffin tissue sections were dewaxed and rehydrated. Antigen retrieval was performed by heating the slides in sodium citrate buffer (10 mM, pH 6.0). After blocking with bovine serum albumin (Sango Biotech, Shanghai, China), the slides were incubated with anti-c-Met (Epitomics, Burlingame, CA, USA), anti-MITF (Cell Signaling Technology, Beverly, MA, USA), or anti-CREB1 (Cell Signaling Technology) overnight at 4 °C. The slides were then incubated with a secondary antibody of goat anti-rabbit HRP conjugate for 1 h at room temperature. A DAB solution was used for brown color development. The strength of positivity was semi-quantified by considering both the intensity and proportion of positive cells.

### Western blot analysis

Western blot analysis was carried out as previously described,^12^ with the following appropriate primary immunoblotting antibodies: anti-GAPDH (Sango Biotech), anti-c-Met, anti-AKT, anti-p-AKT, and anti-fibronectin (Epitomics), and anti-GSK-3*β*, anti-p-GSK-3*β*, anti-Snail, anti-E-cadherin, anti-CREB1, and anti-MITF (Cell Signaling Technology).

### Statistical analysis

The data were expressed as the mean±S.D. Differences between groups were estimated using the *χ*^2^-test or Student's *t*-test. Overall survival rates were calculated according to the Kaplan–Meier method with log-rank test. A Cox regression analysis (proportional hazards model) was performed for the multivariate analyses of prognostic factors. All analyses were performed using SPSS16.0 software (IBM, Armonk, NY, USA) and a two-tailed value of *P*<0.05 was considered statistically significant.

## Abbreviations

miRNA: microRNA
BCa: bladder cancer
EMT: epithelial–mesenchymal transition
3′'-UTR: 3′-untranslated region
qRT-PCR: quantitative real-time PCR
FBS: fetal bovine serum
5-Aza-CdR: 5-Aza-2′-deoxycytidine treatment
BSP: bisulfite-sequencing PCR
IHC: immunohistochemical
TMA: tissue microarray
