# Supplementary material for: c-Met and CREB1 are involved in miR-433-mediated inhibition of the epithelial–mesenchymal transition in bladder cancer by regulating Akt/GSK-3β/Snail signaling
Source: Cell Death Dis. 2016 Feb 4;7(2):e2088–. doi: 10.1038/cddis.2015.274 (PMC4849142; doi:10.1038/cddis.2015.274)
Supplement: Supplementary Table S1 [file cddis2015274x1.doc]

| Table S1. Clinical data of the patients. | | | | |
| --- | --- | --- | --- | --- |
| Patient no. | Sex | Age | TNM stage | Histological grade |
| 1 | Male | 62 | T2N0M0 | III |
| 2 | Male | 60 | T1N0M0 | I |
| 3 | Male | 53 | T1N0M0 | III |
| 4 | Male | 86 | T1N0M0 | III |
| 5 | Male | 55 | T1N0M0 | II |
| 6 | Female | 74 | T2N0M0 | III |
| 7 | Male | 56 | T2N0M0 | III |
| 8 | Female | 76 | T3N0M0 | III |
| 9 | Male | 65 | T2N0M0 | II |
| 10 | Female | 69 | T2N0M0 | II |
| 11 | Male | 72 | T3N0M0 | III |
| 12 | Male | 78 | T1N0M0 | II |
| 13 | Male | 76 | T3N0M0 | III |
