# Supplementary material for: c-Met and CREB1 are involved in miR-433-mediated inhibition of the epithelial–mesenchymal transition in bladder cancer by regulating Akt/GSK-3β/Snail signaling
Source: Cell Death Dis. 2016 Feb 4;7(2):e2088–. doi: 10.1038/cddis.2015.274 (PMC4849142; doi:10.1038/cddis.2015.274)
Supplement: Supplementary Table S3 [file cddis2015274x3.doc]

| Table S3. Multivariate Cox model analysis of overall survival. | | |
| --- | --- | --- |
| **Variables** | **Hazard ratio (95 % CI)** | ***P* value** |
| Sex |  |  |
| Female vs. male | 2.667 (0.678-10.482) | 0.160 |
| Age (years) |  |  |
| >67 vs. ≤67 | 0.847 (0.317-2.260) | 0.740 |
| Grade |  |  |
| High vs. low | 1.086 (0.635-1.858) | 0.763 |
| T stage |  |  |
| T3-T4 vs. Tis-T2 | 1.530 (0.940-2.492) | 0.087 |
| Lymph node metastasis |  |  |
| Positive vs. negative | 4.476 (1.455-13.766) | 0.009 |
| CREB1 |  |  |
| High vs. low | 0.322 (0.110-0.946) | 0.039 |
| c-Met |  |  |
| High vs. low | 1.698 (0.606-4.757) | 0.314 |
