# Supplementary material for: c-Met and CREB1 are involved in miR-433-mediated inhibition of the epithelial–mesenchymal transition in bladder cancer by regulating Akt/GSK-3β/Snail signaling
Source: Cell Death Dis. 2016 Feb 4;7(2):e2088–. doi: 10.1038/cddis.2015.274 (PMC4849142; doi:10.1038/cddis.2015.274)
Supplement: Supplementary Table S4 [file cddis2015274x4.doc]

| Table S4.The oligonucleotides used in this study. | |
| --- | --- |
| **Name^a^** | **Sequence(5’->3’)^b^** |
| miR-433 mimics (sense) | AUCAUGAUGGGCUCCUCGGUGU |
| NC (sense) | ACTACTGAGTGACAGTAGA |
| miR-433 F | ATCATGATGGGCTCCTCGGTGT |
| U6 F | TGCGGGTGCTCGCTTCGGCAGC |
| c-Met F | TGTCCCGAGAATGGTCATAA |
| c-Met R | AGGGAAGGAGTGGTACAACA |
| CREB1 F | ATTCACAGGAGTCAGTGGATAGT |
| CREB1 R | CACCGTTACAGTGGTGATGG |
| MITF F | TTATAGTACCTTCTCTTTGCCAGTCC |
| MITF R | GTTTATTTGCTAAAGTGGTAGAAAGGTACT |
| GAPDH F | AAGGTGAAGGTCGGAGTCA |
| GAPDH R | GGAAGATGGTGATGGGATTT |
| c-Met-utr-wt F | CGTATGAAATAATTTAGTCATCATGAAATATTTAGTTGTCATATAAG |
| c-Met-utr-wt R | TCGACTTATATGACAACTAAATATTTCATGATGACTAAATTATTTCATACGAGCT |
| c-Met-utr -mut F | CGTATGAAATAATTTAGTGTAGTACTAATATTTAGTTGTCATATAAG |
| c-Met-utr-mut R | TCGACTTATATGACAACTAAATATTAGTACTACACTAAATTATTTCATACGAGCT |
| CREB1-utr-wt1 F | CAAATTTTCAACGCCAGGAATCATGAAGAGACTTCTGCTTTTCAACG |
| CREB1-utr-wt1 R | TCGACGTTGAAAAGCAGAAGTCTCTTCATGATTCCTGGCGTTGAAAATTTGAGCT |
| CREB1-utr-wt2 F | CCTTTGAGGGGCTGAACATATCATGAAGCTGAGTCAGTATGGAAAAG |
| CREB1-utr-wt2 R | TCGACTTTTCCATACTGACTCAGCTTCATGATATGTTCAGCCCCTCAAAGGAGCT |
| CREB1-utr-mut F | CCTTTGAGGGGCTGAACATTAGTACTAGCTGAGTCAGTATGGAAAAG |
| CREB1-utr-mut R | TCGACTTTTCCATACTGACTCAGCTAGTACTAATGTTCAGCCCCTCAAAGGAGCT |
| GCLC-utr-wt1 F | CAATAACATATCTAAAGTCATCATGAACTGGCTTGTACATTTTTAAG |
| GCLC-utr-wt1 R | TCGACTTAAAAATGTACAAGCCAGTTCATGATGACTTTAGATATGTTATTGAGCT |
| GCLC-utr-wt2 F | CTATAAAACTCATCAATAAATCATGAAAGGCACTGAGTTTTGTAAAG |
| GCLC-utr-wt2 R | TCGACTTTACAAAACTCAGTGCCTTTCATGATTTATTGATGAGTTTTATAGAGCT |
| TGFBR1-utr-wt1 F | CACTTAAAAGGTGGGATGGATCATGATTACTGTCGATAACTGCAGAG |
| TGFBR1-utr-wt1 R | TCGACTCTGCAGTTATCGACAGTAATCATGATCCATCCCACCTTTTAAGTGAGCT |
| TGFBR1-utr-wt2 F | CAAGATTTGTGAACTGAATATCATGAACCATGTTTTGATACCCCTTG |
| TGFBR1-utr-wt2 R | TCGACAAGGGGTATCAAAACATGGTTCATGATATTCAGTTCACAAATCTTGAGCT |
| ARMC1-utr-wt1 F | CTGCAATTTGCAATAAGTTATCATGAAAAGTTTTTAGATTACACGAG |
| ARMC1-utr-wt1 R | TCGACTCGTGTAATCTAAAAACTTTTCATGATAACTTATTGCAAATTGCAGAGCT |
| ARMC1-utr-wt2 F | CATTTTACTGTGCTTTTCTTCATGAAGGGTACATGCTTTGTACTCTG |
| ARMC1-utr-wt2 R | TCGACAGAGTACAAAGCATGTACCCTTCATGAAGAAAAGCACAGTAAAATGAGCT |
| ARMC1-utr-wt3 F | CTTCTTGAGAGTTAATGTGATCATGATATTGCAAACAACTATAAATG |
| ARMC1-utr-wt3 R | TCGACATTTATAGTTGTTTGCAATATCATGATCACATTAACTCTCAAGAAGAGCT |
| ^a^ F, forward primer; R, reverse primer.  ^b^ Restriction sites are in bold | |
