# Supplementary figures and images for: c-Met and CREB1 are involved in miR-433-mediated inhibition of the epithelial–mesenchymal transition in bladder cancer by regulating Akt/GSK-3β/Snail signaling
Source: Cell Death Dis. 2016 Feb 4;7(2):e2088–. doi: 10.1038/cddis.2015.274 (PMC4849142; doi:10.1038/cddis.2015.274)

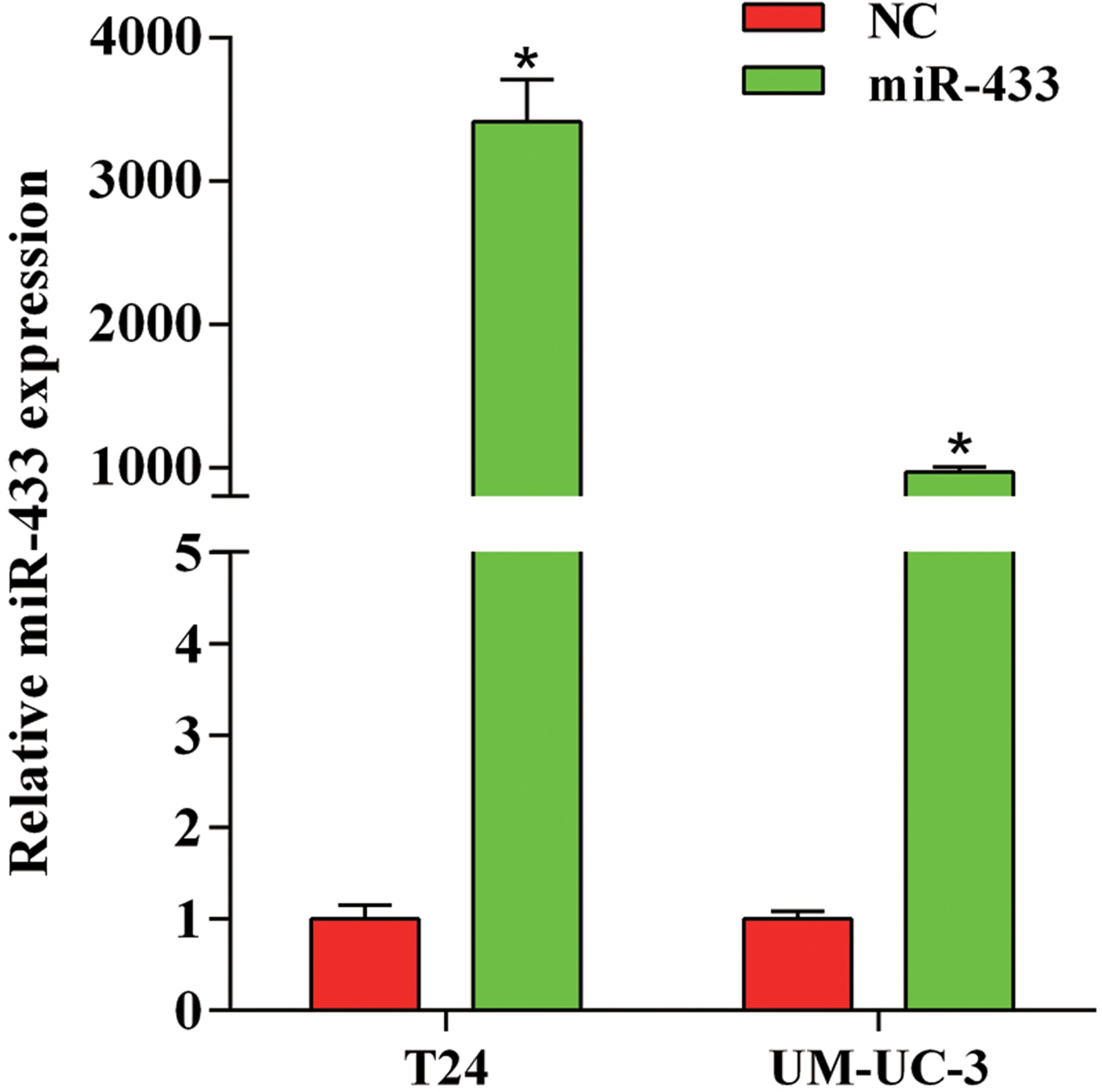

Supplement: Supplementary Figure S1 [file cddis2015274x5.tif]

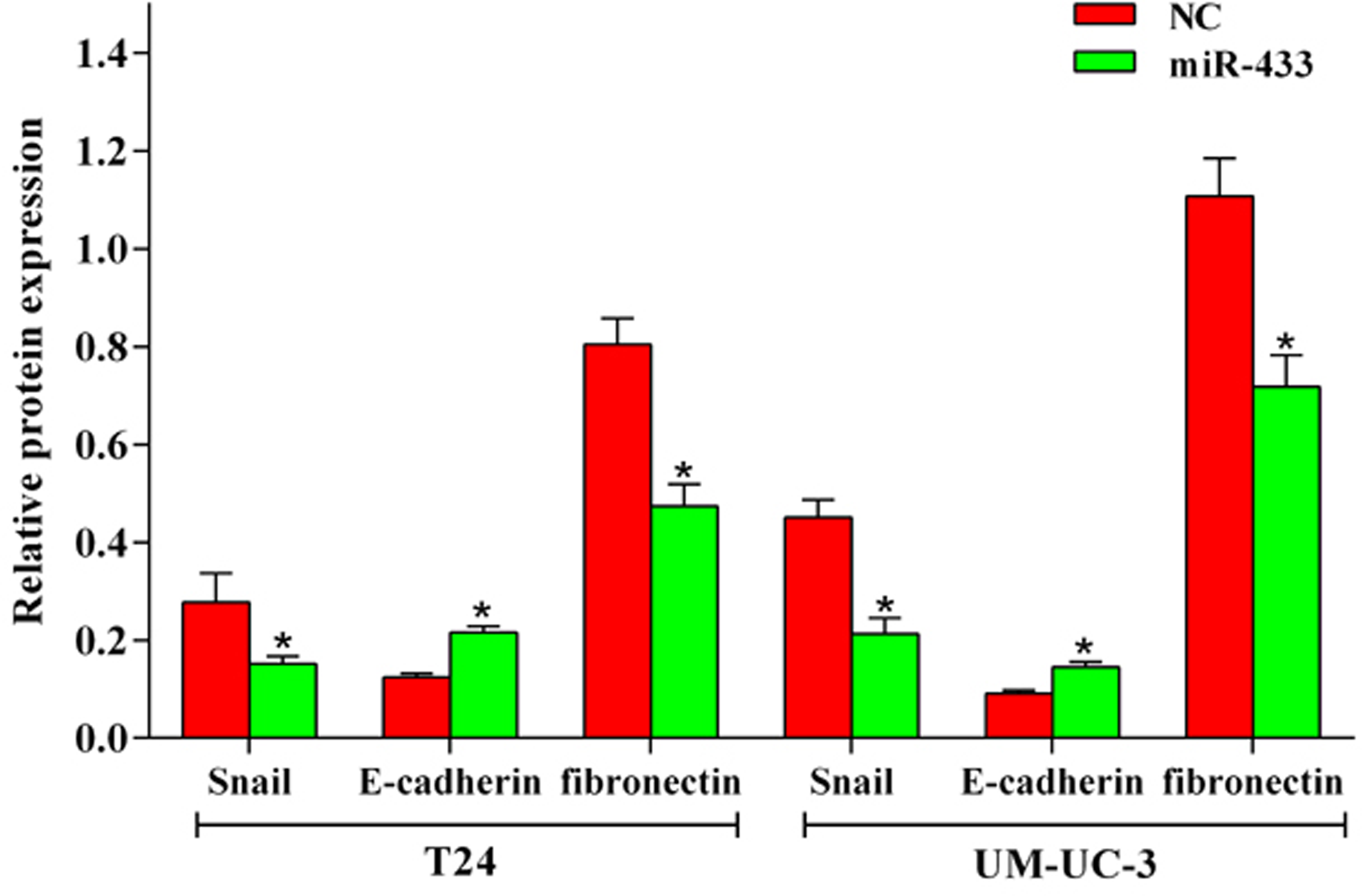

Supplement: Supplementary Figure S2 [file cddis2015274x6.tif]

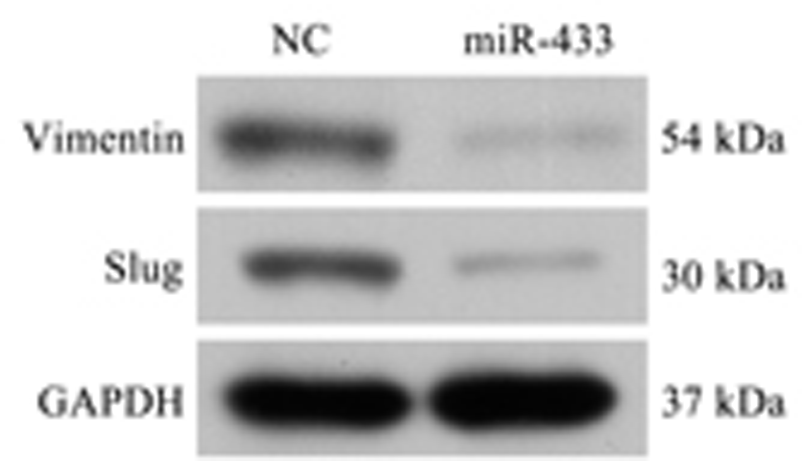

Supplement: Supplementary Figure S3 [file cddis2015274x7.tif]

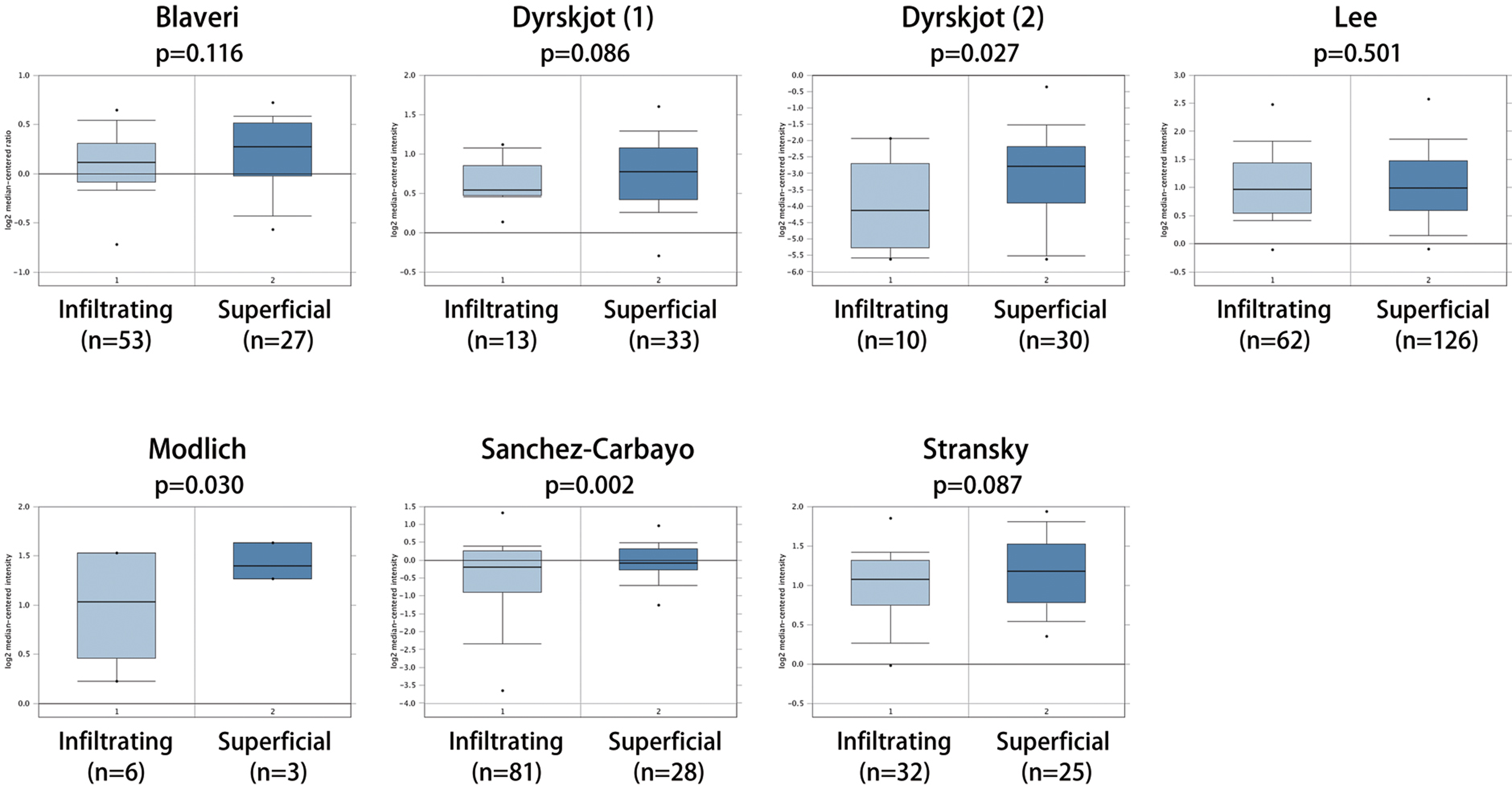

Supplement: Supplementary Figure S4 [file cddis2015274x8.tif]
