# Supplementary material for: c-Met and CREB1 are involved in miR-433-mediated inhibition of the epithelial–mesenchymal transition in bladder cancer by regulating Akt/GSK-3β/Snail signaling
Source: Cell Death Dis. 2016 Feb 4;7(2):e2088–. doi: 10.1038/cddis.2015.274 (PMC4849142; doi:10.1038/cddis.2015.274)
Supplement: Supplementary Figure Legends [file cddis2015274x9.doc]

c-Met and CREB1 are involved in miR-433-mediated inhibition of the epithelial-mesenchymal transition in bladder cancer by regulating Akt/GSK-3β/Snail signaling

Xin Xu, Yi Zhu, Zhen Liang, Shiqi Li, Xianglai Xu, Xiao Wang, Jian Wu, Zhenghui Hu, Shuai Meng, Ben liu, Jie Qin, Liping Xie, Xiangyi Zheng

**Figure captions for Supplementary Figures 1-4**

Figure S1. The ectopic expression of miR-433 was confirmed by qRT-PCR. Error bars represent the standard error obtained from three independent experiments; **P* < 0.05.

Figure S2. Expression levels were quantitated using ImageJ software (Wayne Rashband); GAPDH was used as a loading control. Error bars represent the standard error obtained from three independent experiments; **P* < 0.05.

Figure S3. Western blot analysis. Overexpression of miR-433 (50 nM) inhibited the protein expression of Vimentin and Slug in UM-UC-3 cells.

Figure S4. Analysis of the existing BCa microarray data sets deposited in the Oncomine database. The CREB1 expression in the superficial samples was generally higher than that in the infiltrating samples in 6/7 data sets, of which 3 data sets have statistically significant differences.
